# Patient empowerment in young persons with chronic conditions: Psychometric properties of the Gothenburg Young Persons Empowerment Scale (GYPES)

**DOI:** 10.1371/journal.pone.0201007

**Published:** 2018-07-20

**Authors:** Mariela Acuña Mora, Koen Luyckx, Carina Sparud-Lundin, Mariëlle Peeters, AnneLoes van Staa, Jane Sattoe, Ewa-Lena Bratt, Philip Moons

**Affiliations:** 1 Institute of Health and Care Sciences, University of Gothenburg, Gothenburg, Sweden; 2 KU Leuven Department of Public Health and Primary Care, KU Leuven, Leuven, Belgium; 3 School Psychology and Development in Context, KU Leuven, Leuven, Belgium; 4 Research Centre Innovations in Care, Rotterdam University of Applied Sciences, The Netherlands; 5 Department of Pediatric Cardiology, The Queen Silvia Children’s Hospital, Gothenburg, Sweden; 6 Department of Paediatrics and Child Health, University of Cape Town, South Africa; Public Library of Science, UNITED KINGDOM

## Abstract

**Purpose:**

Empowerment in patients can lead to a higher participation in care and self-management skills. However, there are a limited number of high-quality instruments to assess empowerment and its various dimensions in young persons. The aim was to develop and assess the psychometric properties of the Gothenburg Young Persons Empowerment Scale (GYPES).

**Methods:**

The GYPES is a 15-item questionnaire designed to measure patient empowerment in young persons with chronic conditions. Three studies were conducted to evaluate the psychometric properties of the scale. Studies I and II assessed face, content and factorial validity, as well as responsiveness and reliability in young persons with congenital heart disease and diabetes. After these studies problematic items were identified and reworded and the final version of the GYPES was tested in young persons with diabetes in study III.

**Results:**

The content and face validity of the scale was confirmed in study I. Confirmatory factor analyses (CFA) in study II supported the five-factor structure of the GYPES. However, one item had a low factor loading. The scale was revised and evaluated in study III. CFA of this version supported adequate model fit with factor loadings ranging from 0.385–0.941. A second-order model had an adequate fit to the data. Cronbach’s alpha for the overall scale was 0.858 and for each subscale, alphas range from 0.609–0.858.

**Conclusions:**

GYPES was developed to measure patient empowerment in young persons with chronic conditions. Preliminary evidence supports that the GYPES may be a valid and reliable tool for assessing young persons’ empowerment.

## Introduction

The increase of people living with chronic conditions has compelled society to develop and implement strategies that promote collaboration between patients and healthcare professionals, and which provide the patients with an active role in their care [1, 2]. In order to accomplish this, the World Health Organization, amongst others, has advocated including patient empowerment in healthcare policy development [3]. Patient empowerment is a concept that stems from social sciences. When it was introduced in healthcare, the aim of enhancing patient empowerment was to increase patients’ autonomy and participation in care [4].

In the literature, several definitions and attributes of empowerment are described [4, 5]. Some authors considered it to be: “the capacity individuals have to become responsible for their own health” [6]; “a complex process of change guided by self-determination” [7]; or “a social process where the patients’ abilities to feel in control of their own lives are enhanced” [8]. Even though there are several definitions of empowerment, some of them are not theoretically grounded, or the distinction with other concepts (i.e. self-management) is unclear [7].

Important theoretical developments and empirical underpinnings have been made by Small and co-workers [9]. They defined empowerment as “*an enabling process or outcome arising from communication with the health care professional and a mutual sharing of resources over information relating to illness*, *which enhances the patients’ feelings of control*, *self-efficacy*, *coping abilities and ability to achieve change over their condition*” [9]. Based on qualitative research they also developed a conceptual model proposing that empowerment comprises five dimensions: 1) identity; 2) knowledge and understanding; 3) personal control; 4) shared-decision making; and 5) enabling others (i.e. peers with similar conditions) [9].

A particular group of patients with chronic conditions are young persons with childhood-onset diseases. Patient empowerment is highly relevant in this group because through patient empowerment, young persons with chronic conditions can develop psychosocial skills (e.g. goal-setting, stress management, problem-solving), actively participate in care, and become aware of the need to remain in follow-up [6, 7]. Interventions aiming at increasing the level of patient empowerment have been found to result in improvements in quality of life and well-being [5], better pain management, and increased knowledge of one’s disease [7]. On the longer term, clinical outcomes are considered to be better when patient empowerment is enhanced [5].

To date, there is no universally accepted measure of assessing patient empowerment [10, 11]. A recent literature review identified 19 existing instruments [10]. The problem with most instruments is that they are of low quality, and reliability and validity have not been reported [10, 12]. Moreover, there is no instrument suitable for use in young persons with chronic conditions. For this purpose the Gothenburg Young Persons Empowerment Scale (GYPES) was developed. The aim of the present article is to describe the development of the GYPES and report on consecutive studies in which we evaluated the psychometric properties.

## Methods

### Development of GYPES

GYPES was developed in line with the theoretical foundations of Small and co-workers [9]. In order to avoid response fatigue among the future GYPES respondents, it was important to keep the instrument as brief as possible. To this end three items were generated for each of the five dimensions, resulting in a 15-item questionnaire. Since empowerment is a generic concept applicable to all chronic conditions, we wanted the GYPES to be applicable across the board. For this reasons, the items developed were generic instead of disease-specific.

The development of the GYPES consisted of three stages. During stage one, the involved researchers were divided in two groups. One group (MAM; CSL) developed a set of candidate items based on literature and inspired by existing instruments. The other group (ELB; PM) created a set of potential items based on the definitions of each dimension and on clinical experiences of working with young persons with chronic conditions. The candidate items were formulated in English. In stage two, the two lists were combined into one item pool, comprising 44 potential items. The researchers selected three items that were most representative for each dimension. Sometimes, items were combined and/or reworded to have a good representation of the respective empowerment dimension. In stage three, the 15 items were translated from English to Swedish. A forward translation technique was conducted by two of the authors (CSL, ELB) whose first language is Swedish [13]. These two authors translated the items separately and then discussed the suggested translation until consensus and conceptual equivalence was reached [14]. This developmental process resulted in an English and Swedish version of the GYPES that were subject for further psychometric testing.

### Evaluation of GYPES

Three consecutive studies have been conducted to evaluate the psychometric properties of the GYPES; to optimize the item formulation and to arrive at the final version of the instrument. In Study I, the face and content validity were tested. After revising the wording of some items, Study II was conducted on young persons with congenital heart disease (CHD) in order to evaluate the content validity, factorial validity, internal consistency, and responsiveness of the initial version of the instrument. Some problematic items were identified in this study (see results) so, further optimizations were performed. The final version of the GYPES was tested in Study III, which was conducted on young persons with type 1 diabetes mellitus (DM1).

In order to conduct Study I and II approval was granted from the Regional Ethical Board of Gothenburg, Sweden (No.931-15). According to Swedish regulations, young persons between the ages of 15–18 years are able to give consent in order to participate in studies without need for parental approval. Persons under the age of 15 years require parental approval.

Ethical approval was not required to conduct Study III since in The Netherlands approval by an ethical committee is only required when performing research related to patients treatment or to evaluate healthcare. Questionnaire studies that are not too detailed, burdensome or intimate are exempted from ethics approval. Consent was obtained automatically when patients responded the questionnaires.

### Study I: Face and content validity

#### Sample

Cognitive interviews were conducted with patients from two pediatric cardiology centers and one pediatric outpatient diabetes unit in Sweden. Patients were eligible for these cognitive interviews if they were aged 14–19 years and if they had a scheduled appointment in the participating units in the week that the interviews were held (November-December 2015). Participants were recruited consecutively and comprised 6 patients with CHD (5 girls, 1 boy) and 3 with DM1 (1 girl, 2 boys), with an age range of 14–19 years.

#### Procedure

The participants were asked to fill out the scale together with one of the researchers (ELB, CSL, AB) and to provide feedback on the comprehensibility of the items (probing), response options available and possible suggestions on how to improve the instrument. Interviewers documented the participants’ comments in their field notes. This process helped to identify structural problems in the GYPES and issues with interpretability.

#### Analysis

Data from all interviews were discussed by the interviewers and items were rephrased if the participants stated difficulties in understanding the meaning of an item and/or considered it to be repetitive.

### Study II: Content validity, factorial validity, internal consistency, and responsiveness in CHD

#### Sample

As part of the STEPSTONES project (Swedish Transition Effects Project Supporting Teenagers with chrONic mEdical conditionS), a cross-sectional study was conducted on young persons with CHD in Sweden. The main aim of this cross-sectional questionnaire study was to describe transitional care outcomes such as the level of empowerment, transition readiness, knowledge, health behavior, perceived health status, illness perception and quality of life in adolescents with CHD, and to investigate its correlates. Eligible participants were selected from the Swedish Registry of Congenital Heart Disease (SWEDCON) [15]. Patients were included if they had a confirmed diagnosis of CHD, defined as “*a gross structural abnormality of the heart or the intrathoracic great vessels that is actually or potentially of functional significance*” [16]; if they were aged 14–18 years at the time of the study; and if they were receiving follow-up care at one of the four pediatric centers of CHD in Sweden that participated in the study. Patients were excluded if they had a cognitive and/or physical limitation that did not allow them to answer the questionnaire; had undergone heart transplantation; or had not provided consent to participate.

In all, 593 patients met the inclusion criteria and were sent a set of questionnaires. In total, 202 patients completed and returned the questionnaires, corresponding with a response rate of 34.1%. Demographic characteristics of this sample are presented in Table 1. The mean age of the sample was 15.7 years; 55% were boys. At the time of the study, the majority of participants were in junior high school (57.4%) (Table 1). Participants and non-participants did not differ on sex and disease complexity. There was a significant difference (p<0.05) between the participants (15.7±1.1) and non-participants’ age (15.5±1.1). However, this difference was considered not clinically relevant since the Cohen’s d was 0.18.

**Table 1:** Sample characteristics.

	Study II N = 202 n (%)	Study III N = 273 n (%)
**Sex**		
Male	111 (55)	60 (22.0)
Female	91 (45)	213 (78.0)
**Mean age (standard deviation)**	15.7 (1.2)	19.9 (3.7)
**Education**		
Junior high school	116 (57.4)	42 (15.4)
Senior high school	86 (42.6)	121 (44.3)
College/University	0	110 (40.3)

#### Procedure

Each participant received a package containing information about the study, an informed consent, a set of questionnaires and a pre-addressed envelope. They were asked to return completed questionnaires and the signed informed consent in the pre-addressed envelope. To minimize the non-response rate, a modified Dillman procedure was used [17]. Three weeks after the documents were sent, non-responders received a personalized reminder letter. After five weeks, persistent non-responders received a second reminder letter and a new set of questionnaires. Finally, if participants had not returned the questionnaires after seven weeks, they were contacted by telephone. On this occasion they were asked whether they had received the questionnaires, if their address was correct and whether they wanted to participate in the study. Data collection ran from January 25^th^ till August 31^st^ 2016.

#### Statistical analyses

In Study II, the psychometric evaluation of the GYPES was based on content validity, factorial validity, internal consistency and responsiveness. For content validity, the proportion of missing values and invalid scores was calculated because these parameters indicate how intelligible an item is [18].

Factorial validity was evaluated through confirmatory factor analysis (CFA) to assess the hypothesized factor structure of the scale, i.e. the five dimensions of empowerment. A chi-square index (χ^2^ index) as small as possible, comparative fit index (CFI) value >0.90, a root mean square error of approximation (RMSEA) and standardized root mean square residual (SRMR) <0.08 were used to assess an adequate model fit [19, 20]. After testing a first-order five-factor model, we tested a second-order model with five first-order factors and a second-order global empowerment factor.

Reliability was assessed by calculating the Cronbach’s alpha coefficient as a measure for internal consistency. Coefficients were calculated for the overall scale and for each dimension [21].

Floor and ceiling effects were calculated in order to assess the scale’s potential responsiveness. Floor and ceiling effects were considered present if more than 15% of the participants achieved the lowest or highest score [22, 23].

Statistical analyses were performed with Mplus version 7 software (Muthèn & Muthèn, Los Angeles, CA) for CFA. IBM SPSS Statistics for Windows version 22 was used for other analyses (Armonk, NY: IBM Corp.).

### Study III: Factorial validity, internal consistency, and responsiveness in diabetes

#### Sample

As part of the “Better Transition in Diabetes project”, a cross-sectional study was conducted on young persons with DM1 in the Netherlands. The main aim of the cross sectional study was to describe experiences with transitional care and outcomes, such as level of empowerment, transfer experiences and coping with DM1 in daily life. Eligible participants were members from the *Dutch Diabetes Association* (DVN) and *Stichting ééndiabetes* (a Dutch foundation for young persons with DMI), all aged 12–25 years.

A total of 273 individuals participated in the study. Respondents’ characteristics are shown in Table 1. Three quarters of the patients were female (78.0%). The mean age was 19.9 years and most of the participants were in senior high school (44.3%) or at college or university (40.3%).

#### Procedure

Both patient organizations disseminated the invitation for this survey through their networks and posted a call on their Facebook pages. DVN also sent an email to its members and a reminder after two weeks. *Ééndiabetes* posted a news article about the project on their website. Respondents voluntarily filled out the GYPES using a web-based system. All questionnaires were submitted anonymously and no identifying data was collected from the participants. To encourage participation, five gift vouchers worth €50 were raffled among the respondents. Data collection ran from mid-June to mid-September 2016.

#### Statistical analyses

The same statistical approach has been used as in Study II.

## Results

### Study I: Face and content validity

All participants were able to understand the questions and the response categories. However, three participants with CHD thought the response categories were vague and confusing. Three participants (2 with CHD, 1 with diabetes) said that two items from the “enabling others” dimension were difficult to differentiate and that they needed additional time to understand what they were measuring. One participant with CHD also said that an item in the identity dimension was difficult to understand.

#### Required changes of the instrument

Based on this feedback, we modified some responses of the 5-point Likert scale in the Swedish version in order to increase interpretability of the options.

One item from the “enabling others” dimension was reworded. After these changes, the GYPES resulted in a 15-item instrument measured in a 5-point Likert scale (strongly disagree, disagree, neither agree nor disagree, agree and strongly agree) scale that was later used in Study II.

### Study II: Factorial validity, internal consistency, and responsiveness in congenital heart disease

#### Content validity

The proportion of missing values ranged from 0.5–3.4% (Table 2). Items from the “shared decision-making” and “enabling others” dimensions had the highest missing values (Table 2).

**Table 2:** Missing values and factor loadings for both versions of GYPES.

Factors and items	Missing values n* (%)	Study II^a^ Initial version of GYPES	Study III^b^ Final version of GYPES**
**Knowledge and Understanding**			
1^c^^d^. I know and understand *my condition*	1 (0.5)	0.585	0.706
2 ^c^^d^. I know what to do to stay healthy	1 (0.5)	0.744	0.843
3 ^c^^d^. I know when to contact healthcare providers for *my condition*	4 (2.0)	0.531	0.517
**Personal control**			
4 ^c^^d^. I have the skills to manage *my condition* in daily life	3 (1.5)	0.475	0.762
5 ^c^^d^. I have a sense of control over my health	1 (0.5)	0.876	0.627
6 ^c^^d^. I am active in maintaining my health	2 (1)	0.621	0.385
**Identity**			
7 ^c^. M*y condition* is a part of who I am	2 (1)	0.590	
7 ^d^. *My condition* is a part of who I am as a person			0.460
8 ^c^^d^. Living with *my condition* makes me stronger as a person	1 (0.5)	0.892	0.541
9 ^c^. *My condition* does not stop me from living the life I want to live	1 (0.5)	0.185	
9 ^d^. I have given *my condition* a place in my heart			0.714
**Decision making**			
10 ^c^^d^. I am capable of expressing to my healthcare providers what is important to me	5 (2.5)	0.727	0.679
11 ^c^. I actively participate in discussions about my health	1 (0.5)	0.609	
11 ^d^. I actively participate in discussions with my health care providers about my health			0.869
12 ^c^^d^. I am capable of making decisions about my health and health care together with the healthcare providers	6 (3.0)	0.773	0.772
**Enabling others**			
13 ^c^^d^. I have the skills to support other young people with *my condition*	7 (3.4)	0.761	0.783
14 ^c^. I feel comfortable sharing with others about *my condition*	4 (2.0)	0.531	
14 ^d^. I am able to give helpful advice to people who are struggling with their *condition*			0.941
15. I can help other people by sharing how I keep myself well	4 (2.0)	0.808	0.649

^a^ Five factor model

^b^ Five factor model with error correlation between factors

^c^ Congenital heart disease

^d^ Diabetes

*Number of missing values per item of participants with CHD who answered at least one item of the scale

** No missing values per item were found for participants of this study

All factor loadings were significant at p<0.0001, except for item 9 in Study II (p = 0.014)

#### Factorial validity

The aim of the CFA was to test a five-factor structure, in accordance with the five dimensions of empowerment. Based on the fit indices (*d*ƒ: 80; χ^2^: 154.948, p<0.0001; CFI: 0.916; RMSEA: 0.068; SRMR: 0.069), the model had an adequate fit (Table 3). Factor loadings are shown in Table 2. The factor loadings of the items varied between 0.475 and 0.892, apart from the identity factor, which had one item with a low factor loading (0.185, p = 0.014). If this item was deleted and a new model with the five-factor structure with only two items in the “identity” dimension was run, an improved model fit was observed (see Table 3). We also evaluated the second-order factor model but the model did not converge properly, which may be due to including only two items in the “identity” dimension.

**Table 3:** Model fit statistics in confirmatory factor analysis.

Fit index	Study II Initial version of GYPES		Study III Final version of GYPES		
	Model 1^a^	Model 2^b^	Model 1^a^	Model 2^c^	Model 3^d^
Comparative fit index (CFI)	0.916	0.930	0.897	0.919	0.908
Root mean square error of approximation (RMSEA)	0.068	0.067	0.084	0.076	0.078
Standardized root mean square residual (SRMR)	0.069	0.058	0.059	0.057	0.061
Chi-square test of model fit • Degrees of Freedom • P-value • Normed chi^2^ index (x^2^/df)	154.948 80 <0.0001 1.937	128.464 67 <0.0001 1.917	235.375 80 <0.0001 2.942	201.950 79 <0.0001 2.556	222.788 84 <0.0001 2.652

^a^ Five factor confirmatory analysis.

^b^ Five factor confirmatory analysis without item 9.

^c^ Five factor confirmatory analysis with error correlation between factors.

^d^ Five factor confirmatory analysis with error correlation and second order factor.

#### Internal consistency

Cronbach’s alpha coefficients for all factors were above 0.6, apart from identity, which had a value of 0.521 (Table 4). The value for the overall scale was of 0.819, reflecting an adequate level of internal consistency. The mean total empowerment score was 54.5 ± 10.5 on a scale from 15 to 75.

**Table 4:** Cronbach’s alpha values.

Factors	Study II^a^ Initial version of GYPES	Study III^b^ Final version of GYPES
Knowledge and Understanding	0.633	0.693
Personal control	0.672	0.636
Identity	0.521	0.609
Shared decision-making	0.751	0.806
Enabling others	0.707	0.833
Overall scale	0.819	0.858

^a^ Congenital heart disease

^b^ Diabetes

#### Responsiveness

None of the patients had the lowest possible score and only 1.5% of the participants had the highest possible score, indicating there were no floor or ceiling effects.

#### Required changes in the instrument

We have modified some items of the version of the GYPES used in Study II on statistical and substantive grounds. For statistical reasons, item 9 was rephrased because it turned out to be a problematic item in the “identity” dimension. For substantive reasons, we rephrased items 7 (identity), item 11 (shared decision-making) and item 14 (enabling others) to further improve consistency and increase understanding.

The revised version of GYPES was translated from English into Dutch following a forward-backward translation process [24]. Two translators (one bilingual in Dutch and English) translated the instrument independently from each other and presented the results to the Dutch team. The translation was tested for face validity with two mothers and a 16-year old adolescent with epilepsy. Consensus was reached after discussing both versions. This process resulted in the final version of the GYPES that was evaluated in Study III (Additional File 1: Final version of the GYPES).

### Study III: Factorial validity, internal consistency, and responsiveness in diabetes

#### Content validity

No missing values were found for the GYPES items, suggesting that the wording of the items was intelligible.

#### Factorial validity

**Fig 1:**
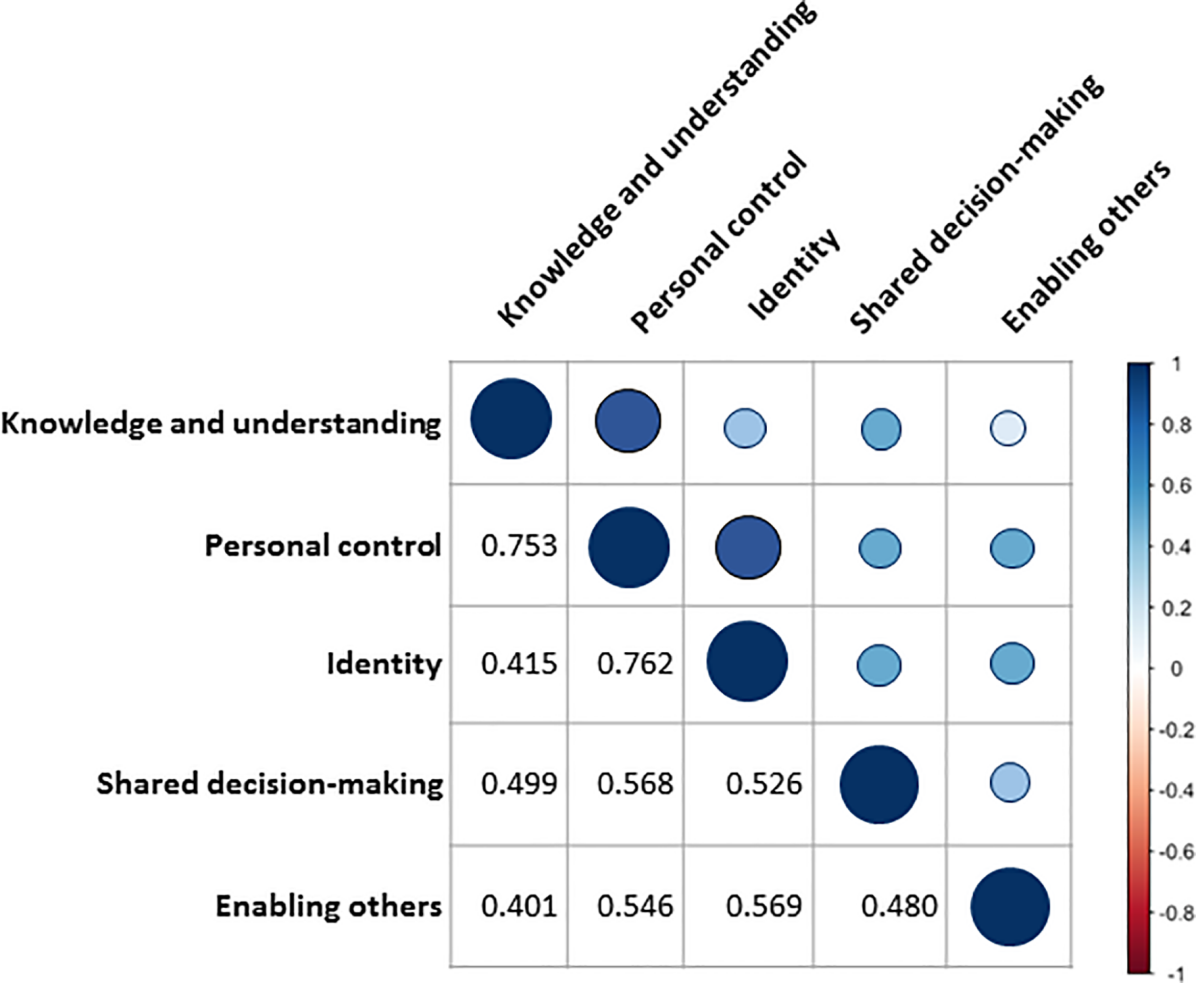
Latent correlations within factors.

CFA was also used to examine if the five-factor structure fitted with the observed variables. Fit indices generally indicated an adequate model, although CFI did not reach the threshold of 0.90 (*d*ƒ: 80; χ^2^: 235.375, p<0.0001; CFI: 0.897; RMSEA: 0.084; SRMR: 0.059) (Table 3). In order to improve model fit based on the modification indices we evaluated a second model allowing one error correlation between factors (item 6 with 15). This was the largest error correlation and even when these items belong to different dimensions, it can be theoretically expected that individuals who are more actively involved in their care, feel more capable of sharing their experiences and coping techniques with other individuals [25]. This showed an adequate fit across all indices (*d*ƒ: 79; χ2: 201.950, p<0.0001; CFI: 0.919; RMSEA: 0.076; SRMR: 0.057) with factor loadings that ranged from 0.385 to 0.941 (Table 3). The latent factor correlations of this model are shown in Fig 1.

Moreover, a third model assessing the second-order factor model showed an adequate fit as well (*d*ƒ: 84; χ^2^: 222.788, p<0.0001; CFI: 0.908; RMSEA: 0.078; SRMR: 0.061). The items’ factor loadings for this model had values from 0.379 to 0.938. The factor loadings of each first-order factor in relation to the global empowerment factor had values exceeding 0.660 (knowledge and understanding: 0.729; shared decision-making: 0.660; personal control: 0.950; identity: 0.777; and enabling others: 0.617). Results from this model support the calculation of subscale scores, as well as an overall empowerment score.

#### Internal consistency

The final version of the GYPES showed to be internally consistent with a Cronbach’s alpha of 0.858. Alpha values were acceptable for all the subscales, with values above 0.6 (Table 4). The mean empowerment score in this sample was 58.9±7.9 on a scale from 15 to 75.

#### Responsiveness

None of the participants had the lowest scoring and only 2.2% of the participants had the highest possible score. Thus, the GYPES is not subject to floor or ceiling effects.

## Discussion

Involving persons with chronic conditions in their care and improving clinical and patient outcomes can be achieved through enhancing patient empowerment. To date, there are limited number of high-quality measurements for this construct [10], and an instrument to be used in young persons with chronic conditions did not exist. Consequently, we developed the GYPES based on theoretical and empirical grounds [9] and evaluated the psychometric properties in an iterative way.

Such an empowerment scale is relevant for transitional care and research. For instance, it can be used to assess the impact of empowerment-enhancing interventions in young persons with chronic conditions [26].

### Validity

The studies have provided preliminary evidence on the validity of GYPES to measure the level of empowerment in young persons with chronic conditions. Indeed, we found evidence for the content validity and structural validity of the instrument. Respondents of the two clinical populations understood the items and response categories well, and the theoretically grounded five-dimensional structure has been confirmed. The second-order factor model demonstrated that in addition to the subscale scores, an overall empowerment score can be validly computed.

Given these findings GYPES has several advantages over other measurements, which lack subscales clarifying the dimensions behind this construct [27–29], or which measure empowerment as a subscale of a broader concept [30, 31]. Moreover, most existing instruments are meant to be used in one specific patient population [28, 32, 33]. Although we provided initial evidence on the content and structural validity of GYPES, we were not able to investigate other aspects of validity such as criterion-related or construct validity. This is to be taken up in future studies.

### Reliability

Cronbach’s alpha coefficients for each of the subscales and the total scale were acceptable, which supports the internal consistency of GYPES. Even more, the alpha values are relatively high considering that each subscale is comprised of only three items. These findings indicate that subscale and total scores can be reliably calculated. Stability of the instruments was not assessed in the present studies. Future studies with longitudinal designs should take this up. In this matter, suitable techniques ought to be used to distinguish between the stability of the construct and the stability of the instrument [34].

### Responsiveness

Responsiveness is a critical, although often neglected attribute of questionnaires. It refers to the capacity to detect clinically meaningful changes over time [35]. Because the current studies were cross-sectional, we were not able to scrutinize responsiveness. However, as a proxy, we assessed a potential floor and ceiling effect because such effects jeopardize the responsiveness of instruments. We found no indication that GYPES have floor or ceiling effects.

### Methodological issues

The present studies have several methodological strengths: (i) we developed the GYPES based on theoretical and empirical grounds and evaluated the psychometric properties in an iterative way; (ii) we included respondents from distinct clinical populations and living in different countries, which increases generalizability; (iii) we involved the target population during content validity testing as a way of increasing the readability and comprehension of the scale [36]; and (iv) we applied CFA techniques, which are more powerful than exploratory factor analyses that is traditionally used.

However, there were also some limitations; (i) Study II had a low response rate, which may have led to a response bias; (ii) the sample in Study III was self-selected, which increases the possibility that the sample is not representative of the target population; (iii) the sample in study III was older than in Study II which could affect the interpretation of the items. This could also explain why there was a significant difference between the total empowerment scores in Studies II and III; (iv) some aspects of validity and reliability were not addressed, which should be taken up in future research; and (v) although there were intensive discussions between the researchers regarding the translations into Swedish, we did not apply a forward-backward translation by two independent translators.

### Conclusion

We developed the GYPES as an instrument to measure the level of empowerment in young persons with chronic conditions. The GYPES is a 15-item instrument, covering five dimensions of empowerment. We have provided preliminary evidence on the validity and reliability of the scale. The GYPES can be used in descriptive studies to depict the level of empowerment in patients, or in intervention studies to evaluate the impact of healthcare interventions on patient empowerment.

## Supporting information

S1 File GYPES-CHD Swedish version. (PDF)

S2 File GYPES-CHD English version. (PDF)

S3 File GYPES-Diabetes data. (XLSX)

S4 File GYPES-CHD data. (XLSX)

S5 File Dutch guidelines for questionnaire research. (PDF)

## Abbreviations

CFA: confirmatory factor analysis
CFI: comparative fit index
CHD: congenital heart disease
DM1: type 1 diabetes mellitus
DVN: Dutch Diabetes Association
GYPES: Gothenburg Young Persons Empowerment Scale
RMSEA: root mean square error of approximation
STEPSTONES: Swedish Transition Effects Project Supporting Teenagers with chrONic mEdical conditionS
SRMR: standardized root mean square residual
SWEDCON: Swedish Registry of Congenital Heart Disease
