# Supplementary material for: Patient empowerment in young persons with chronic conditions: Psychometric properties of the Gothenburg Young Persons Empowerment Scale (GYPES)
Source: PLoS One. 2018 Jul 20;13(7):e0201007. doi: 10.1371/journal.pone.0201007 (PMC6054395; doi:10.1371/journal.pone.0201007)
Supplement: S1 File — (PDF) [file pone.0201007.s001.pdf]

Gothenburg Young Persons Empowerment Scale – Congenital Heart Disease (GYPES-CHD v1.1 – Svenska)

Instruktion: Sätt ett kryss i den ruta du känner stämmer bäst med din situation

		Instämmer inte alls	Instämmer delvis	Varken eller	Instämmer mestadels	Instämmer helt och hållet
1	Jag känner till och förstår mitt hjärtfel	☐	☐	☐	☐	☐
2	Jag vet vad jag skall göra för att må bra	☐	☐	☐	☐	☐
3	Jag vet när jag ska kontakta sjukvården på grund av mitt hjärtfel	☐	☐	☐	☐	☐
4	Jag har förmåga att hantera mitt hjärtfel i dagligt liv	☐	☐	☐	☐	☐
5	Jag känner att jag har kontroll över min hälsa	☐	☐	☐	☐	☐
6	Jag arbetar aktivt för att behålla min hälsa	☐	☐	☐	☐	☐
7	Mitt hjärtfel är en del av mig som person	☐	☐	☐	☐	☐
8	Att leva med mitt hjärtfel gör mig starkare som person	☐	☐	☐	☐	☐
9	Mitt hjärtfel är en del av mitt liv	☐	☐	☐	☐	☐
10	Jag kan uttrycka det som är viktigt för mig till vårdpersonalen	☐	☐	☐	☐	☐
11	Jag deltar aktivt i diskussioner om min hälsa med vårdpersonalen	☐	☐	☐	☐	☐
12	Jag kan besluta om min hälsa och sjukvård tillsammans med vårdpersonalen	☐	☐	☐	☐	☐
13	Jag känner att jag har förmågan att ge stöd till andra unga personer med medfödda hjärtfel	☐	☐	☐	☐	☐
14	Jag kan ge råd till personer som upplever att de har problem på grund av sitt hjärtfel	☐	☐	☐	☐	☐
15	Jag kan hjälpa andra genom att dela med mig om hur jag gör för att må bra	☐	☐	☐	☐	☐

INSPECTION COPY - DO NOT USE

Developed by Bratt EL, Sparud-Lundin C, Acuña Mora M, Moons P. 2016 (© EL Bratt, 2016)
Permission for the use of the GYPES can be obtained by email: Ewa-Lena.Bratt@gu.se Philip.Moons@gu.se
