# Supplementary material for: Patient empowerment in young persons with chronic conditions: Psychometric properties of the Gothenburg Young Persons Empowerment Scale (GYPES)
Source: PLoS One. 2018 Jul 20;13(7):e0201007. doi: 10.1371/journal.pone.0201007 (PMC6054395; doi:10.1371/journal.pone.0201007)
Supplement: S2 File — (PDF) [file pone.0201007.s002.PDF]

Gothenburg Young Persons Empowerment Scale – Congenital Heart Disease (GYPES-CHD v1.1 – English)

Instruction: Put an “X” in the appropriate boxes that best fit your situation.

		Strongly disagree	Disagree	Neither agree, nor disagree	Agree	Strongly agree
1	I know and understand my heart disease	☐	☐	☐	☐	☐
2	I know what to do to stay healthy	☐	☐	☐	☐	☐
3	I know when to contact health care providers for my heart disease	☐	☐	☐	☐	☐
4	I have the skills to manage my heart disease in daily life	☐	☐	☐	☐	☐
5	I have a sense of control over my health	☐	☐	☐	☐	☐
6	I am active in maintaining my health	☐	☐	☐	☐	☐
7	My heart disease is part of who I am as a person	☐	☐	☐	☐	☐
8	Living with my heart disease makes me stronger as a person	☐	☐	☐	☐	☐
9	I have given my heart disease a place in my life	☐	☐	☐	☐	☐
10	I am capable of expressing to my healthcare providers what is important to me	☐	☐	☐	☐	☐
11	I actively participate in discussions with my healthcare providers about my health	☐	☐	☐	☐	☐
12	I am capable of making decisions about my health and health care with the healthcare providers	☐	☐	☐	☐	☐
13	I have the skills to support other young people with a heart disease	☐	☐	☐	☐	☐
14	I am able to give helpful advice to people who are struggling with their heart disease	☐	☐	☐	☐	☐
15	I can help other people by sharing how I keep myself well	☐	☐	☐	☐	☐

INSPECTION COPY - DO NOT USE

Developed by Bratt EL, Sparud-Lundin C, Acuña Mora M, Moons P. 2016 (© EL Bratt, 2016)
Permission for the use of the GYPES can be obtained by email: Ewa-Lena.Bratt@gu.se Philip.Moons@gu.se
