# Supplementary material for: Patient empowerment in young persons with chronic conditions: Psychometric properties of the Gothenburg Young Persons Empowerment Scale (GYPES)
Source: PLoS One. 2018 Jul 20;13(7):e0201007. doi: 10.1371/journal.pone.0201007 (PMC6054395; doi:10.1371/journal.pone.0201007)
Supplement: S5 File — (PDF) [file pone.0201007.s005.PDF]

Questionnaire research

Research which requires filling in a questionnaire just once generally does not fall under the scope of the Medical Research Involving Human Subjects Act (WMO).

However, if you plan on asking the research subjects questions which are **detailed**, **burdensome**, or are **intimate**, for example of a sexual or psychological nature? Or will it take the research subject a lot of time to fill in the questionnaire? Then the research may fall under the scope of the WMO.

When in doubt contact your MREC. More information on the reviewing of WMO research can be found at Review procedure. This website also contains information on non-WMO research.
